# Screening of Reference Genes for RT-qPCR in Chicken Adipose Tissue and Adipocytes

**DOI:** 10.3389/fphys.2021.676864

**Published:** 2021-05-14

**Authors:** Wei Na, Yuxiang Wang, Pengfei Gong, Xinyang Zhang, Ke Zhang, Hui Zhang, Ning Wang, Hui Li

**Affiliations:** ^1^Key Laboratory of Chicken Genetics and Breeding, Ministry of Agriculture and Rural Affairs, Harbin, China; ^2^Key Laboratory of Animal Genetics, Breeding and Reproduction, Education Department of Heilongjiang Province, Harbin, China; ^3^College of Animal Science and Technology, Northeast Agricultural University, Harbin, China; ^4^College of Animal Science and Technology, Hainan University, Haikou, China

**Keywords:** reference gene, RT-qPCR, adipocytes, adipose tissue, broilers

## Abstract

Reverse transcription quantitative real-time PCR is the most commonly used method to detect gene expression levels. In experiments, it is often necessary to correct and standardize the expression level of target genes with reference genes. Therefore, it is very important to select stable reference genes to obtain accurate quantitative results. Although application examples of reference genes in mammals have been reported, no studies have investigated the use of reference genes in studying the growth and development of adipose tissue and the proliferation and differentiation of preadipocytes in chickens. In this study, GeNorm, a reference gene stability statistical algorithm, was used to analyze the expression stability of 14 candidate reference genes in the abdominal adipose tissue of broilers at 1, 4, and 7 weeks of age, the proliferation and differentiation of primary preadipocytes, as well as directly isolated preadipocytes and mature adipocytes. The results showed that the expression of the TATA box binding protein (*TBP*) and hydroxymethylbilane synthase (*HMBS*) genes was most stable during the growth and development of abdominal adipose tissue of broilers, the expression of the peptidylprolyl isomerase A (*PPIA*) and *HMBS* genes was most stable during the proliferation of primary preadipocytes, the expression of the *TBP* and *RPL13* genes was most stable during the differentiation of primary preadipocytes, and the expression of the *TBP* and *HMBS* genes was most stable in directly isolated preadipocytes and mature adipocytes. These results provide reference bases for accurately detecting the mRNA expression of functional genes in adipose tissue and adipocytes of chickens.

## Introduction

Gene expression analysis is an important tool in investigating functional genes. Due to the advantages of simple operation, high sensitivity and good repeatability, reverse transcription quantitative real-time PCR (RT-qPCR) has become the main method to detect gene expression levels (Bustin, 2000; Dheda et al., 2005). To reduce background errors caused by mRNA quality and reverse transcription efficiency (Lekanne Deprez et al., 2002; Sanders et al., 2014), correction factors are usually applied to correct, and standardize experimental results (Huggett et al., 2005; Hendriks-Balk et al., 2007). The most commonly used correction factor is the reference gene. Reference genes are genes with stable expression levels that are not affected by research conditions and can be used to accurately quantify initial material loads (Dong et al., 2013). The ideal reference genes are stably expressed in all kinds of tissues and cells, and their expression is not influenced by the environment, experimental conditions, or other factors (de Jonge et al., 2007). However, a universal ideal reference gene does not exist (Huggett et al., 2005; Zhang et al., 2014). Even the most versatile reference genes, such as glyceraldehyde-3-phosphate dehydrogenase (*GAPDH*), beta-actin (*ACTB*) and 18S ribosomal RNA (*18S*), are unstable in certain cells, biological processes, or experimental conditions (Barroso et al., 1999; Bas et al., 2004; Ferguson et al., 2010; Stephens et al., 2011). Therefore, the selection of suitable reference genes that are stably expressed in a specific tissue, cell or biological process is very important to accurately quantify the expression level of functional genes.

The stability of reference genes generally requires verification by experiments combined with algorithm analysis. GeNorm is a statistical algorithm developed by Vandesompele et al. (2002) to analyze the expression stability of reference genes in RT-qPCR experiments. The algorithm determines stably expressed reference genes by comparing the *M* value which is defined the average of the pairwise variation of one gene with all the other potential reference genes. The geNorm algorithm can be used to compare and filter the stability of any number of reference genes under any experimental conditions. Additionally, the geNorm algorithm can determine the number of optimal reference genes in the experiment by calculating the ratio of paired variant *V*_*n*_ to *V*_*n*__+__1_.

At present, the literature on the screening of RT-qPCR reference genes has focused on chicken liver, brain, muscle, heart, lung, ovaries, lymphoid organs, and chicken embryo fibroblasts (Yang et al., 2013; Bagés et al., 2015; Nascimento et al., 2015; Mitra et al., 2016; Staines et al., 2016; Katarzyńska-Banasik et al., 2017; Hassanpour et al., 2018; Simon et al., 2018). No reports have selected RT-qPCR reference genes in the growth and development of abdominal adipose tissue, primary preadipocytes, or mature adipocytes of chickens. Therefore, based on the usage frequency in the literatures on the screening of RT-qPCR reference genes in various tissues and cells of chickens and function of reference genes in the basic cellular processes, 14 commonly used reference genes were proposed as candidates: *ACTB*, tubulin beta class I (*TUBB*), hypoxanthine guanine phosphoribosyl transferase 1 (*HPRT1*), hydroxymethylbilane synthase (*HMBS*), TATA box binding protein (*TBP*), non-POU domain containing, octamer-binding (*NONO*), ribosomal protein L13 (*RPL13*), ribosomal protein S7 (*RPS7*), *18S*, peptidylprolyl isomerase A (*PPIA*), beta-2 microglobulin (*β2M*), tyrosine 3-monooxygenase/tryptophan 5-monooxygenase activation protein zeta (*YWHAZ*), *GAPDH*, and transferrin receptor (*TFRC*). The expression levels of these reference genes in broiler abdominal adipose tissue, the proliferation and differentiation of primary preadipocytes, as well as directly isolated primary preadipocytes and mature adipocytes were detected by RT-qPCR. The expression stability of these reference genes in the above tissues and cells was analyzed by GeNorm statistical algorithms. Finally, we confirmed the most suitable reference genes for detecting functional gene expression levels in broiler adipose tissue and adipocytes.

## Materials and Methods

### Ethics Statement

The study was conducted according to the guidelines for the care and use of experimental animals established by the Ministry of Science and Technology of the People’s Republic of China (approval no. 2006–398) and approved by the Institutional Biosafety Committee of Northeast Agricultural University (Harbin, China).

### Animals and Tissues

The animals used in this study included 1-, 4-, and 7-week-old birds from generation 19 (G19) of Northeast Agricultural University broiler lines divergently selected for abdominal fat content (NEAUHLF; Guo et al., 2011). All birds were housed under similar environmental conditions with free access to feed and water.

The abdominal fat tissues from each individual male bird were collected and snap-frozen in liquid nitrogen and stored at −80^°^C for extraction of total RNA. A total of 30 male birds (five birds per line per time point) at 1, 4, and 7 weeks of age were used in this process.

### Isolation of Chicken Preadipocytes and Mature Adipocytes

Chicken preadipocytes and mature adipocytes were isolated according to previously reported methods (Wang et al., 2017). Briefly, abdominal adipose tissue was collected from 10- to 14-day-old broilers by sterile dissection following rapid decapitation. Adipose tissue was washed with prewarmed PBS and cut into pieces. Tissue pieces were digested in medium (90 mL DMEM, 1.5 g BSA, and 2.383 g HEPES) containing type I collagenase (0.02 g/mL) for 65 min at 37^°^C with shaking once per 5 min. After digestion, the cell suspension was filtered through a 165-μm mesh and centrifuged at 2,000 rpm for 10 min at room temperature. The supernatant was collected and centrifuged at 4,000 rpm; the obtained cell precipitates were mature adipocytes. The protocell precipitate was resuspended in complete medium (DMEM/F12, 10% fetal bovine serum, 100 U/mL penicillin, and 100 μg/ml streptomycin) and filtered through a 20-μm mesh. After centrifugation at 2,000 rpm, the precipitates of stromal vascular cells (including preadipocytes) were collected. Some of the preadipocytes were used for gene expression analysis, some of which were seeded at a density of 1 × 10^6^ cells/mL in complete medium and maintained in a humidified atmosphere with 5% CO_2_ at 37^°^C for cell proliferation and differentiation detection.

### Induced Chicken Preadipocyte Differentiation

When the confluence of chicken preadipocytes reached 50–60%, culture medium was replaced with an induction medium in which the final concentration of oleate was 160 μM to induce preadipocyte differentiation. This time was recorded as 0 h. The differentiated preadipocytes were harvested at the following times:12, 24, 48, 72, and 96 h. Three wells of cells were collected at each time point for biological repeats. In the induction process, the induction medium was changed every 24 h.

### Staining and Measurement of Lipid Droplet

Lipid droplets were stained with oil red O (Sigma) according to Wang et al. (2017), with some modifications. Briefly, the induced chicken preadipocytes were washed with PBS and fixed with 10% (v/v) formalin at 4^°^C for 30 min. After washing again with PBS, the cells were stained with oil red O staining solution (oil red O solution: deionized water = 3:2) for 30 min. Then, the cells were immediately washed with ddH_2_O and observed and photographed under an inverted fluorescence microscope (Leica).

To accurately quantify lipid droplet accumulation in preadipocytes, an oil red O extraction assay was used. In short, after staining with oil red O, the preadipocytes were washed with PBS. Then, 1 mL 100% (v/v) isopropanol was added to extract oil red O and measured at 510 nm using a plate reader (Synergy H1, BioTek Instruments, Inc, United States).

### Correction of Total Cell Protein

The collected cells were added to 150 μL RIPA lysis solution containing 1 mM phenylmethanesulfonyl fluoride (PMSF; Biyuntian) and stirred until there was no cell mass. After lysis for 30 min on ice, the cell solution was centrifuged at 12,000 rpm for 10 min, and the supernatant was taken as total cell protein. The total protein concentration was determined using a bicinchoninic acid kit (Biyuntian). The absorbance value of the oil red O extraction/total protein amount is due to oil red O colorimetry after protein correction.

### Detection of Chicken Preadipocyte Proliferation

A CCK-8 assay was used to detect chicken preadipocyte proliferation. When the confluence of the passaged chicken primary preadipocytes reached 30, 50, 70, 90, and 100%, the cells were added to a 10% (v/v) CCK-8 solution (DOJINDO) and incubated for 4 h. Then, the absorbance value of the colored medium was measured at 450 nm by a plate reader (Synergy H1, BioTek Instruments, Inc, United States). Three wells of cells were collected at each time point for biological repetition.

### Total RNA Extraction and cDNA Synthesis

Total RNA from tissues and cells was extracted by TRIzol reagent (Invitrogen) following manufacturer instructions. RNA quality was determined by ultraviolet spectrophotometry (Eppendorf) and electrophoresis. Only total RNA of the best quality and purity (absorbance ratio of OD260 to OD280 between 1.8 and 2.2) was intended for further analysis. First-strand cDNA synthesis was performed with 1 μg total RNA using a PrimeScript RT reagent Kit (TaKaRa) and stored at −20^°^C.

### Primer Design and RT-qPCR

In this study, 14 reference genes were selected according to the literature for screening reference genes in chicken tissues and cells as well as their function and usage frequency. The basic information and primer sequences of the 14 reference genes are shown in Tables 1, 2, respectively. Quantitative real-time RT-PCR was performed to detect the expression level of these genes on a QuantStudio^TM^ Real-time PCR system (Applied Biosystems). The reaction procedure was as follows: 1 cycle at 95^°^C for 10 min, followed by 40 cycles of 95^°^C for 15 s and 60^°^C for 1 min, and a melting curve analysis (95^°^C for 15 s, 60^°^C for 1 min, and 95^°^C for 15 s). After amplification, the dissociation curves for each PCR were analyzed using Dissociation Curve 1.0 software (Applied Biosystems) to detect and eliminate possible primer dimer artifacts.

**TABLE 1:** Information of the 14 reference genes.

**Gene symbol**	**Full name**	**Function**
*ACTB*	Actin, beta	Cytoskeletal structural protein
*TUBB*	Tubulin beta class I	Major constituent of microtubules
*HPRT1*	Hypoxanthine guanine phosphoribosyl transferase 1	Purine synthesis in salvage pathway
*HMBS*	Hydroxymethylbilane synthase	Heme synthesis pathway
*TBP*	TATA box binding protein	Basal transcription machinery
*NONO*	non-POU domain containing, octamer-binding	Regulation of transcription
*RPL13*	Ribosomal protein L13	60S ribosomal protein L13
*RPS7*	Ribosomal protein S7	40S ribosomal protein S7
*18S*	18S ribosomal RNA	Cytosolic small ribosome subunit, translation
*PPIA*	Peptidylprolyl isomerase A (cyclophilin A)	Peptidyl-prolyl *cis*-trans isomerase activity
*β2M*	Beta-2 microglobulin	MHC class I molecules. Defense/immunity protein
*GAPDH*	Glyceraldehyde-3-phosphate dehydrogenase	Glycolysis
*YWHAZ*	Tyrosine 3-monooxygenase/tryptophan 5-monooxygenase activation protein zeta	Signal transduction
*TFRC*	Transferrin receptor (p90, CD71)	Cellular uptake of iron

**TABLE 2:** Primer sequences of 14 selected internal control genes.

**Gene**	**Accession number**	**Exon (*F*)**	**Exon (*R*)**	**Length**	**Sequence (5′>3′)**
*ACTB*	NM_205518	3	4	142 bp	F: TGAACCCCAAAGCCAACAGAG R: TCACCAGAGTCCATCACAATACCA
*TUBB*	NM_205315	3	4	156 bp	F: GGTAAATATGTGCCACGAGCC R: CTCCGTGTAGTGCCCTTTGG
*HPRT1*	NM_204848	7	9	157 bp	F: TTGTTGGTCAAAAGAACTCCTCG R: TCTGCTTCCCCGTCTCACTG
*HMBS*	XM_417846	11	12	118 bp	F: TCGTGCCAAAGACCAAGAAAC R: GACACTACAGCCACCCTCCAA
*TBP*	NM_205103	4.5	6	122 bp	F: GCGTTTTGCTGCTGTTATTATGAG R: TCCTTGCTGCCAGTCTGGAC
*NONO*	NM_001031532	6	7	151 bp	F: AGAAGCAGCAGCAAGAAC R: TCCTCCATCCTCCTCAGT
*RPL13*	NM_204999	3	4	108 bp	F: TCGTGCTGGCAGAGGATTCA R: GATTTGTTTCTTCGCCTGGGAT
*RPS7*	XM_001234708	5	6	157 bp	F: CTGCCCAAGCCAACGAGAA R: GCCTGCTGCCATCCAGTTTTA
*18S*	AF173612			96 bp	F: CTCTTTCTCGATTCCGTGGGT R: CATGCCAGAGTCTCGTTCGT
*PPIA*	NM_001166326	1	2	91 bp	F: CCAACCCCGTCGTGTTCTTC R: GTTATGGGCACCTTGTCAGCG
β*2M*	NM_001001750	3	4	115 bp	F: ATCCCGAGTTCTGAGCTGTGC R: CCGTCATACCCAGAAGTGCGAT
*YWHAZ*	NM_001031343	5	6	85 bp	F: AGTCATACAAAGACAGCACGCTA R: GCTTCATCTCCTTGGGTATCCGA
*TFRC*	NM_205256	10	10.11	137 bp	F: ATCGGTATGTTGTGATTGGAGCC R: CCTCGGTTTGTAGCCCTCGTT
*GAPDH*	NM_204305	8	9	134 bp	F: CAGAACATCATCCCAGCGTCC R: CGGCAGGTCAGGTCAACAAC

The standard curves of each gene were drawn as follows: first, the cDNA of all samples were mixed together as an initial template, and five gradient cDNA samples were obtained according to a dilution ratio of 5 times the concentration. Second, the concentration of the initial template was defined as 5^0^, and the concentrations of the five gradient cDNA samples were named 5^0^, 5^–1^, 5^–2^, 5^–3^, and 5^–4^. Finally, the five gradient cDNA samples were used as templates for qPCR, and a standard curve was prepared. The accuracy of the PCR products was determined by gel electrophoresis and sequencing.

### Data Analysis

Based on the correlation coefficient *R*^2^ and slope of the standard curve, the amplification efficiency of each pair of reference gene primers was calculated by the following formula: amplification efficiency (%) = (10^(1/–*slope)*^-1) × 100. The GeNorm algorithm was used to compare the *M* value to determine the stability of the reference gene. A smaller *M* value indicates higher stability, and a larger *M* value indicates lower stability. To test the minimum number of reference genes needed for adequate data normalization, geNorm calculates a pairwise variation (*V*) between using *n* (number) and *n* + 1 reference genes (Vandesompele et al., 2002). The ratio of *Vn*/*Vn* + 1 was introduced as a criterion and the default threshold is 0.15, which means the ratio of *Vn*/*Vn* + 1 is greater than 0.15, the most suitable internal reference number is *n* + 1. The original Ct value obtained by RT-qPCR was converted into relative quantitative data by the 2^–Δ*Ct*^ method, ΔCt = Ct_*sample*_-Ct_*min*_. The difference between groups was analyzed using unpaired Student’s *t*-test. Comparisons with *P* < 0.05 and *P* < 0.01 were considered significant and extremely significant, respectively.

## Results and Analysis

To detect internal gene expression levels more accurately, we first analyzed the specificity of 14 pairs of primers. The results showed that the amplification curves and melting curves of 14 pairs of reference gene primers were all clear and single (Supplementary Figures 1, 2). The standard curve correlation coefficients *R*^2^ of 14 reference genes were all greater than 0.99, and the amplification efficiency of primer pairs were 92.943 to 99.76% (Supplementary Table 1), which confirmed that the 14 pairs of primers met the quality control standard of RT-qPCR and could be used for follow-up analyses.

### Screening of RT-qPCR Reference Genes in Broiler Abdominal Adipose Tissue

#### Expression Levels Analysis of 14 Reference Genes in Broilers Abdominal Adipose Tissue

We measured the expression levels of 14 reference genes in abdominal adipose tissue of fat and lean line broilers at the ages of 1, 4, and 7 weeks by RT-qPCR. According to the average Ct value of reference genes, the average expression levels of 14 reference genes in broiler abdominal adipose tissues were divided into three categories: high abundance expression, medium abundance expression, and low abundance expression. The *18S* reference gene, with an average Ct value of 6.59, was high abundance expression. *ACTB, RPL13, RPS7, PPIA, β2M*, and *GAPDH*, with average Ct values of 17.04 to 19.60, were reference genes expressed in medium abundance. *TUBB, HMBS, TBP, NONO, TFRC, HPRT1*, and *YWHAZ*, with average Ct values of 20.44 to 24.75, were reference genes with low abundance expression (Table 3).

**TABLE 3:** The average Ct values of the 14 reference genes in the adipose tissue of broilers.

**Gene**	**The average Ct values**	**Gene**	**The average Ct values**
*ACTB*	17.07 ± 0.88	*PPIA*	19.60 ± 0.68
*TUBB*	21.94 ± 0.57	*β2M*	18.66 ± 0.86
*HMBS*	22.40 ± 0.75	*GAPDH*	18.18 ± 0.97
*TBP*	23.36 ± 0.81	*TFRC*	24.75 ± 0.70
*NONO*	21.53 ± 0.54	*HPRT1*	21.31 ± 0.66
*RPL13*	17.61 ± 0.69	*18S*	6.59 ± 0.29
*RPS7*	17.04 ± 0.75	*YWHAZ*	20.44 ± 0.89

*ACTB, beta-actin; *TUBB*, tubulin beta class I; *HMBS*, hydroxymethylbilane synthase; *TBP*, TATA box binding protein; *NONO*, non-POU domain containing, octamer-binding; *RPL13*, ribosomal protein L13; *RPS7*, ribosomal protein S7; *PPIA*, peptidylprolyl isomerase A; *β2M*, beta-2 microglobulin; *GAPDH*, glyceraldehyde-3-phosphate dehydrogenase; *TFRC*, transferrin receptor; *HPRT1*, hypoxanthine guanine phosphoribosyl transferase 1; *18S*, 18S ribosomal RNA; and *YWHAZ*, yrosine 3-monooxygenase/tryptophan 5-monooxygenase activation protein zeta.*

#### Expression Stability Analysis of 14 Reference Genes in Broiler Abdominal Adipose Tissue

**FIGURE 1:**
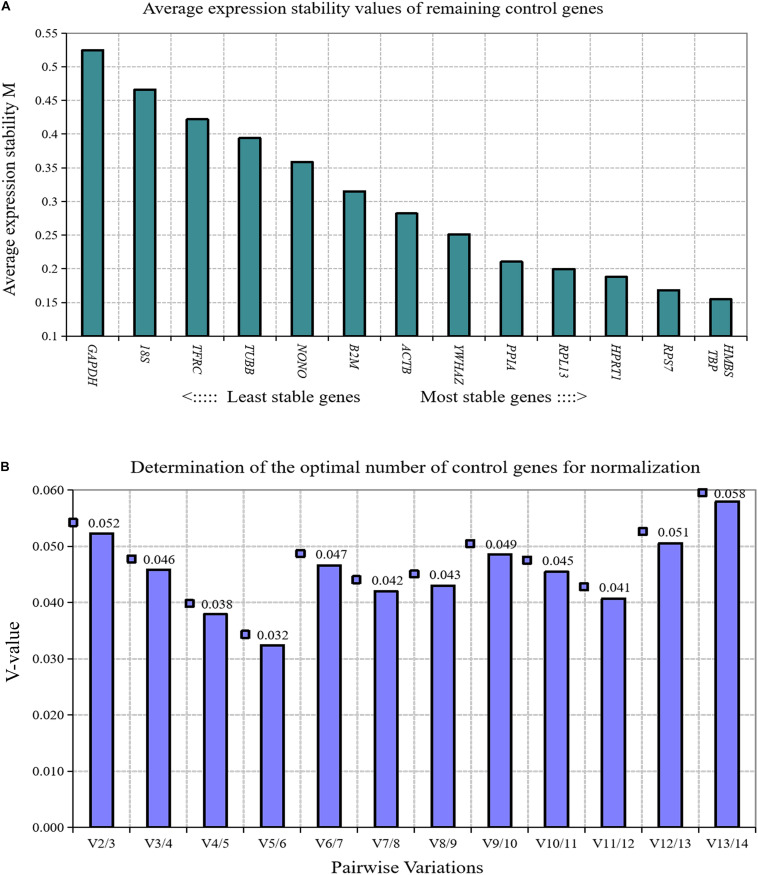
GeNorm analysis of 14 reference genes in broiler abdominal adipose tissue. **(A)** Expression stability analysis. The most stably expressed genes have lower *M* values. *TBP* and *HMBS* were the two best reference genes; **(B)** Pairwise variation analysis. Determination of the optimal number of reference genes required for normalization calculated by pairwise variation analysis between normalization factors NF*_*n*_* and NF*_*n*_*_+__1_. According to geNorm algorithm, two reference genes were needed.

We analyzed the expression stability of these 14 reference genes during the growth and development of abdominal adipose tissue of broilers using GeNorm algorithm. The results showed that the expression stability of 14 reference genes from high to low was *TBP* and *HMBS*, *RPS7, HPRT1, RPL13, PPIA, YWHAZ, ACTB, β2M, NONO, TUBB, TFRC, 18S*, and *GAPDH* (Figure 1A). Paired variation analyses of the reference genes showed that *V*_2/3_ was 0.052, which was less than the recommended threshold of 0.15, indicating that two reference genes were needed as standardized correction factors in the RT-qPCR experiment with broiler abdominal fat tissue as the experimental material (Figure 1B).

### Screening of RT-qPCR Reference Genes in Directly Isolated Primary Preadipocytes and Mature Adipocytes

#### Expression Level Analysis of 14 Reference Genes in Directly Isolated Primary Preadipocytes and Mature Adipocytes

We measured the expression levels of 14 reference genes in directly isolated primary preadipocytes and mature adipocytes. Each kind of cell contained five samples, and every sample came from mixed cells of three chickens. According to the average Ct value of genes, the average expression levels of 14 reference genes in adipocytes can be divided into three categories: high abundance expression, medium abundance expression and low abundance expression. The *18S* gene, with an average Ct value of 6.73, was a reference gene with high abundance expression. *ACTB, RPL13, RPS7, PPIA, GAPDH*, and *YWHAZ*, with average Ct values of 16.80 to 19.86, were reference genes expressed in medium abundance. *TUBB, HMBS, TBP, NONO, β2M, TFRC*, and *HPRT1*, with average Ct values of 20.25 to 25.15, were reference genes with low abundance expression (Table 4).

**TABLE 4:** The average Ct values of the 14 reference genes in chicken primary preadipocytes and mature adipocytes.

**Gene**	**The average Ct values**	**Gene**	**The average Ct values**
*ACTB*	16.80 ± 0.64	*PPIA*	19.06 ± 0.68
*TUBB*	20.47 ± 0.57	*β2M*	20.25 ± 0.86
*HMBS*	22.62 ± 0.80	*GAPDH*	17.98 ± 0.77
*TBP*	23.33 ± 0.82	*TFRC*	25.15 ± 0.60
*NONO*	21.44 ± 0.59	*HPRT1*	20.73 ± 0.86
*RPL13*	17.76 ± 0.74	*18S*	6.73 ± 0.33
*RPS7*	17.78 ± 0.69	*YWHAZ*	19.86 ± 0.76

*ACTB, beta-actin; *TUBB*, tubulin beta class I; *HMBS*, hydroxymethylbilane synthase; *TBP*, TATA box binding protein; *NONO*, non-POU domain containing, octamer-binding; *RPL13*, ribosomal protein L13; *RPS7*, ribosomal protein S7; *PPIA*, peptidylprolyl isomerase A; *β2M*, beta-2 microglobulin; *GAPDH*, glyceraldehyde-3-phosphate dehydrogenase; *TFRC*, transferrin receptor; *HPRT1*, hypoxanthine guanine phosphoribosyl transferase 1; *18S*, 18S ribosomal RNA; and *YWHAZ*, yrosine 3-monooxygenase/tryptophan 5-monooxygenase activation protein zeta.*

#### Expression Stability Analysis of 14 Reference Genes in Directly Isolated Primary Preadipocytes and Mature Adipocytes

**FIGURE 2:**
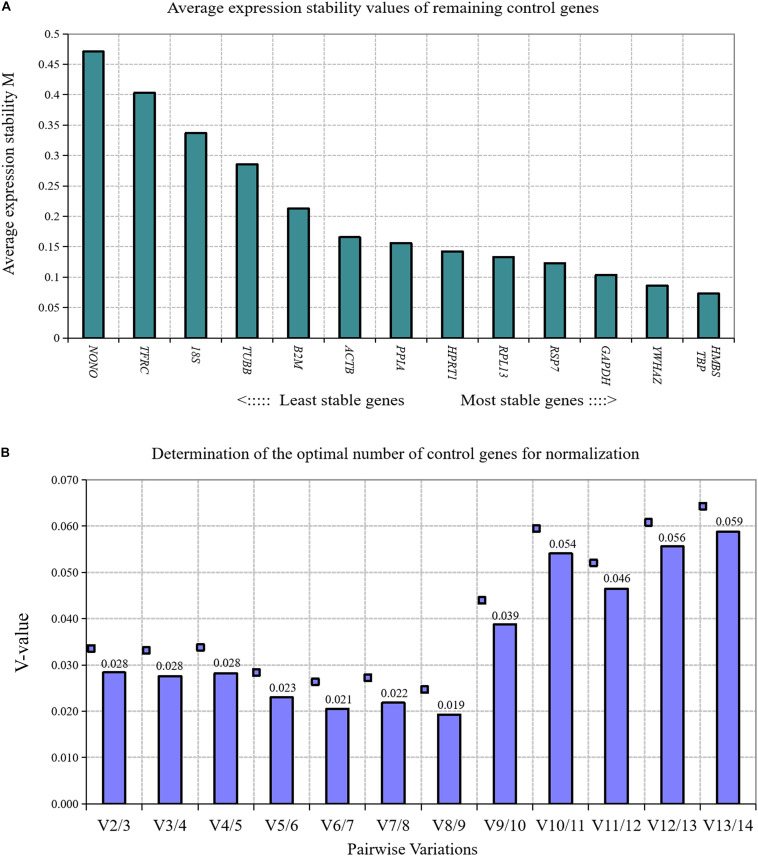
GeNorm analysis of 14 reference genes in directly isolated primary preadipocytes and mature adipocytes. **(A)** Expression stability analysis. The most stably expressed genes have lower *M* values. *TBP* and *HMBS* were the two best reference genes; **(B)** Pairwise variation analysis. Determination of the optimal number of reference genes required for normalization calculated by pairwise variation analysis between normalization factors NF*_*n*_* and NF*_*n*_*_+__1_. According to geNorm algorithm, two reference genes were needed.

We used GeNorm algorithm to analyze the expression stability of 14 reference genes in directly isolated primary preadipocytes and mature adipocytes. The results showed that the stability of 14 reference genes in directly isolated chicken primary preadipocytes and mature adipocytes from high to low was *TBP* and *HMBS, YWHAZ, GAPDH, RPS7, RPL13, HPRT1, PPIA, ACTB, β2M, TUBB, 18S, TFRC*, and *NONO* (Figure 2A). Paired variation analyses of reference genes showed that *V*_2/3_ was 0.028, which was less than the recommended threshold of 0.15, indicating that two reference genes were needed as standardized correction factors in the RT-qPCR experiment with chicken primary preadipocytes and mature adipocytes as the experimental materials (Figure 2B).

### Screening of RT-qPCR Reference Genes in the Differentiation Process of Chicken Primary Preadipocytes

#### Differentiation Detection of Chicken Primary Preadipocytes

**FIGURE 3:**
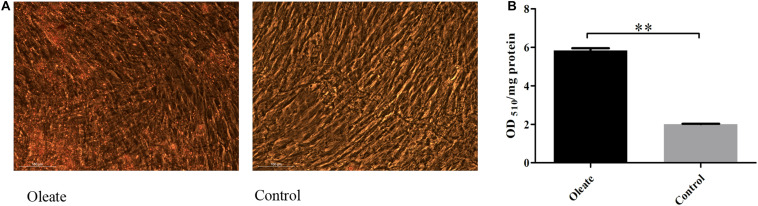
Oil red O staining and content of oil red O at 96 h of induced differentiation. **(A)** Oil red O staining at 96 h of induced differentiation. **(B)** The content of oil red O at 96 h of induced differentiation. ^∗∗^extremely significant (*P* < 0.01).

When primary preadipocytes were induced to differentiate at 96 h, oil red O staining and extraction assays were performed to determine the differentiation status of preadipocytes in the induced group (oleic acid group) and the noninduced group (control group; Figure 3A). The results showed that the lipid deposition of cells in the induction group was significantly higher than that in the control group (*P* < 0.01; Figure 3B), indicating that chicken preadipocytes were successfully induced to differentiate.

#### Expression Stability Analysis of RT-qPCR Reference Genes During Chicken Primary Preadipocyte Differentiation

**FIGURE 4:**
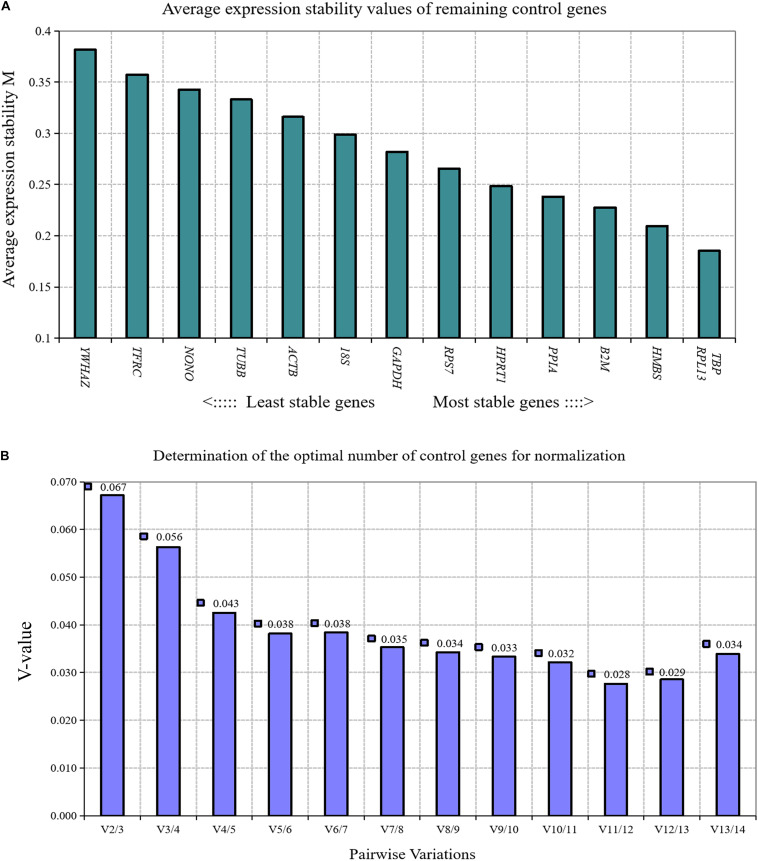
GeNorm analysis of 14 reference genes during chicken primary preadipocyte differentiation. **(A)** Expression stability analysis. The most stably expressed genes have lower *M* values. *TBP* and *RPL13* were the two best reference genes; **(B)** Pairwise variation analysis. Determination of the optimal number of reference genes required for normalization calculated by pairwise variation analysis between normalization factors NF*_*n*_* and NF*_*n*_*_+__1_. According to geNorm algorithm, two reference genes were needed.

GeNorm algorithm was applied to analyze the expression stability of 14 reference genes during chicken primary preadipocyte differentiation (0, 12, 24, 48, 72, and 96 h). The results showed that the expression stability of 14 reference genes during the differentiation of chicken preadipocytes from high to low was *TBP* and *RPL13, HMBS, β2M, PPIA, HPRT1, RPS7, GAPDH, 18S, ACTB, TUBB, NONO, TFRC*, and *YWHAZ* (Figure 4A). Paired variation analyses of the reference gene showed that the value of *V*_2/3_ was 0.067, which was less than the recommended threshold of 0.15, indicating that two reference genes were needed as standardized correction factors in RT-qPCR experiments in the process of inducing the differentiation of chicken primary preadipocytes (Figure 4B).

### Screening of RT-qPCR Reference Genes in the Process of Chicken Primary Preadipocyte Proliferation

#### Detection of the Proliferation Status of Chicken Primary Preadipocytes

**FIGURE 5:**
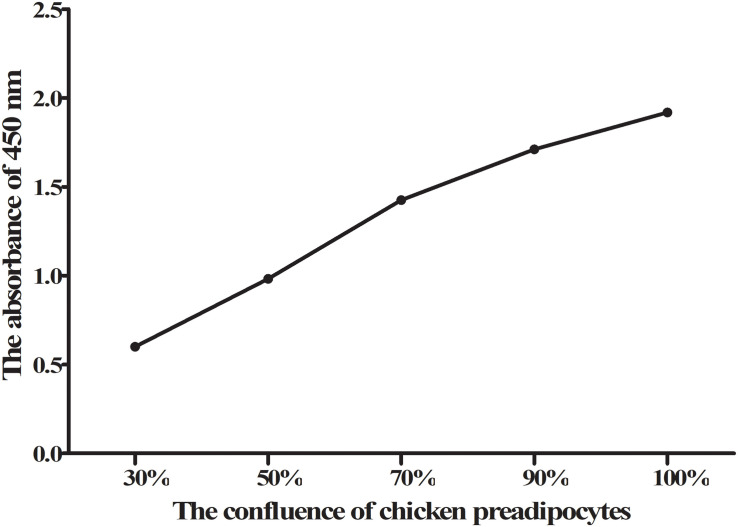
CCK-8 results of chicken primary preadipocyte proliferation. The absorbance of 450 nm gradually increased with the confluence of chicken primary preadipocytes increased, which showed that the primary preadipocytes of chicken were in a normal proliferative state.

When the confluence of chicken primary preadipocytes reached 30, 50, 70, 90, and 100%, the proliferation status of chicken preadipocytes was measured by the CCK-8 method, and a cell growth curve was prepared. The results showed that the primary preadipocytes of chickens were in a normal proliferative state (Figure 5).

#### Expression Stability Analysis of RT-qPCR Reference Genes During the Proliferation of Chicken Primary Preadipocytes

**FIGURE 6:**
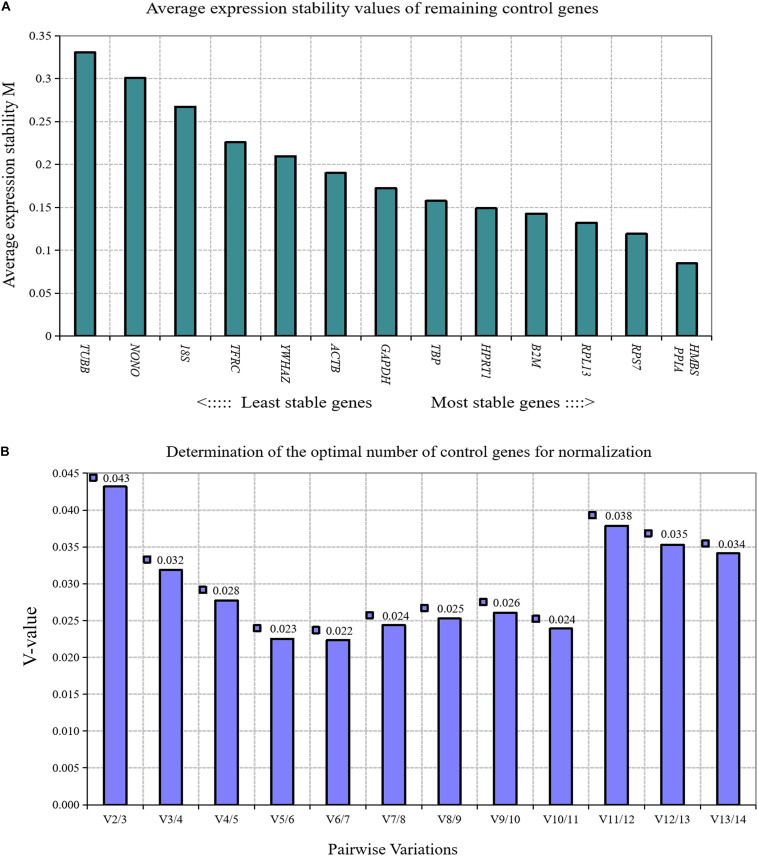
GeNorm analysis of 14 reference genes during the proliferation of chicken primary preadipocytes. **(A)** Expression stability analysis. The most stably expressed genes have lower *M* values. *HMBS* and *PPIA* were the two best reference genes; **(B)** Pairwise variation analysis. Determination of the optimal number of reference genes required for normalization calculated by pairwise variation analysis between normalization factors NF*_*n*_* and NF*_*n*_*_+__1_. According to geNorm algorithm, two reference genes were needed.

GeNorm algorithm was used to analyze the expression stability of 14 reference genes in the proliferation process of chicken primary preadipocytes; cell confluence was 30, 50, 70, 90, and 100%. The expression stability of the 14 reference genes in chicken preadipocytes proliferation process from high to low was *PPIA* and *HMBS, RPS7, RPL13, β2M, HPRT1, TBP, GAPDH*, *ACTB, YWHAZ, TFRC, 18S, NONO*, and *TUBB* (Figure 6A). Paired variation analyses of reference genes showed that the value of *V*_2/3_ was 0.043, which was less than the recommended threshold of 0.15, indicating that two reference genes were needed as standardized correction factors for RT-qPCR data calculation in the proliferation process of chicken preadipocytes (Figure 6B).

## Discussion and Conclusion

Gene expression analysis plays an important role in understanding the complex network of gene regulation. Commonly used gene expression detection methods include semi-quantitative PCR, Northern blot, Western blot, and sequencing. Although these methods can be used for gene expression analysis, they have different degrees of disadvantages, such as low accuracy of semi-quantitative PCR, high complexity of Northern blot and Western blot methods, and high cost of sequencing. Real-time fluorescence quantitative PCR has become the most widely used technology to detect gene expression due to its high accuracy, good sensitivity, and strong specificity (Bustin, 2000; Dheda et al., 2005). At present, there are two main methods for RT-qPCR to determine gene expression levels: absolute quantitative and relative quantitative. An accurate expression level of genes can be obtained by the absolute quantitative method, but different standard samples are required for different genes, which greatly increases the difficulty and complexity of RT-qPCR experiments. By contrast, the relative quantitative method is simpler, more accurate and efficient and is more widely used. In the relative quantitative detection of RT-qPCR, it is usually necessary to select the corresponding reference genes for calibration and standardization (Huggett et al., 2005; Hendriks-Balk et al., 2007). Therefore, the correct application of reference genes directly affects the accuracy of experimental results (Radoniæ et al., 2004). In Bustin et al. (2009) developed a minimum standard of experimental information for the publication of quantitative PCR experimental data. While aimed at improving the reliability of RT-qPCR experimental results, this guide also emphasized the importance of reference gene selection. A valid reference gene should be constitutively and equally expressed in different experimental conditions, tissues or physiological state of the tissue or organism (Kozera and Rapacz, 2013). However, such ideal reference gene is difficult to find. To search reliable reference genes and accurately evaluate the expression stability of these genes, a variety of algorithms, such as GeNorm (Vandesompele et al., 2002), NormFinder (Andersen et al., 2004), and BestKeeper (Pfaffl et al., 2004), have been applied. GeNorm is the most commonly used procedure for calculating a normalization factor based on multiple control genes. This algorithm can calculate the *M* value, which indicates the stability of reference gene expression for a given sample. The higher the *M* value is, the worse the stability of gene expression; in contrast, the lower the *M* value is, the better the stability of gene expression (Vandesompele et al., 2002).

To date, some reference genes have been validated for the standardization of RT-qPCR data in chickens, but these genes are only concentrated in the gene expression analysis of a certain tissue or cell, such as muscle, brain, abdominal fat, heart, lung, ovary, uterus, lymphoid organ, articular cartilage, chicken embryo fibroblasts, IEL-NK cell, and DT40 cell line (Yang et al., 2013; Bagés et al., 2015; Nascimento et al., 2015; Mitra et al., 2016; Staines et al., 2016; Katarzyńska-Banasik et al., 2017; Hassanpour et al., 2018, 2019; Simon et al., 2018; Boo et al., 2020; Dunislawska et al., 2020; Hul et al., 2020; Marciano et al., 2020). Whether these reference genes are also suitable to the study of gene expression during the growth and development of a specific tissue or cell in chickens, such as adipose tissue and adipocyte, still needs to be further determined. In this study, abdominal adipose tissue of broilers at different ages of weeks, primary preadipocytes and mature adipocytes were used as materials to carry out RT-qPCR reference gene screening experiments. This addresses a literature gap on RT-qPCR reference gene screening from the tissue level to the cell level of broilers and provides a reference for the selection of RT-qPCR reference genes in adipose tissue and adipocytes of broilers. The results of this study showed that the *TBP* and *HMBS* genes were stably expressed during the growth and development of broiler abdominal adipose tissue, the *TBP* and *RPL13* genes were stably expressed during the differentiation of chicken primary preadipocytes, the *PPIA* and *HMBS* genes were stably expressed in the proliferation process of chicken primary preadipocytes, and the *TBP* and *HMBS* genes were stably expressed in directly isolated preadipocytes and mature adipocytes. Among them, the *TBP* and *HMBS* genes were stably expressed in many growth and development processes of adipose tissue and cells of broilers, indicating that they can be widely used in the study of gene expression in chickens.

TATA box binding protein can bind to TBP binding factor (TBP-associated factors, TAFs) and combine into transcription factor IID to participate in the transcriptional initiation of genes (Li et al., 2015; Malecova et al., 2016). It has been reported that *TBP* is an reference gene stably expressed in many tissues of chickens in RT-qPCR experiments. Mitra et al. (2016) identified *TBP* as an reference gene stably expressed in spleen, liver, caecum, and caecum tonsil tissues of laying hens using GeNorm, NormFinder, BestKeeper, ΔCt, and RefFinder methods. Simon et al. (2018) analyzed the expression stability of 10 reference genes in the hypothalamus of chickens under three different nutritional conditions using BestKeeper, GeNorm, NormFinder, ΔCt, and a multivariate linear mixed-effect modeling algorithm, and found that *TBP* was one of the most stable reference gene in the hypothalamus of chickens. Bagés et al. (2015) and Hassanpour et al. (2019) also identified *TBP* as an stably expressed reference gene in chicken muscle, liver, ovary, and uterus tissue using GeNorm, NormFinder, CV, and BestKeeper methods. HMBS, the third enzyme in the heme biosynthetic pathway, catalyzes the head-to-tail condensation of four molecules of porphobilinogen to form the linear tetrapyrrole 1-hydroxymethylbilane (HMB; Bung et al., 2018). *HMBS* has been identified as a stably expressing reference gene in many tissues of chickens and is also a commonly used reference gene in chicken RT-qPCR experiments (Cedraz de Oliveira et al., 2017; Lenart et al., 2017; Hassanpour et al., 2019; Boo et al., 2020). Note that only male birds were used in the present study for the selection procedure requirement of the NEAUHLF (Guo et al., 2011). Meanwhile, only juvenile animals were used to more accurately observe the growth and development of abdominal fat tissue in current research. Therefore, our results cannot confirm that *TBP* and *HMBS* genes are still stably expressed during the growth and development of adipose tissue in female birds and adult individuals.

Ribosomal protein L13 is a component of ribosomes and participates in the translation process. The results of this study showed that *RPL13* was stably expressed during the differentiation of chicken primary preadipocytes, which was consistent with the results of human studies. Gentile et al. (2016) showed that ribosomal protein *RPL13A* is the most suitable reference gene to detect the regularity of gene expression during the differentiation of human vascular interstitial cells into adipocytes. *RPL13* also was determined as one of the stable genes in ovary and uterus of laying hens (Hassanpour et al., 2019). PPIA, a multifunctional protein, is the main cytoplasmic binding protein of the immunosuppressive drug cyclosporine A. It has the molecular chaperone activity of peptidyl propyl *cis*-trans isomerase and plays important roles in protein folding, transport, assembly, immunomodulation, and cellular signal transduction (Xu et al., 2010). This study showed that the expression of *PPIA* was stable during the proliferation of chicken primary preadipocytes and relatively stable during the differentiation of primary preadipocytes, which is consistent with the results in mice: the expression of the *PPIA* gene is relatively stable during the induction of differentiation of mouse 3T3-L1 cells (Zhang et al., 2014). In addition, previous research has shown that the expression of *PPIA* in avian brain tissues and gonads is relatively stable (Zinzow-Kramer et al., 2014). These results indicate that the *RPL13* and *PPIA* genes are more suitable for the study of gene expression levels during the proliferation and differentiation of chicken adipocytes.

In addition, when applying RT-qPCR technology for gene expression analysis, it is often necessary to select multiple reference genes as calibration standards to obtain more reliable results. While analyzing the stability of gene expression, GeNorm algorithm can also calculate the paired variation *V* value of the standardized factor after introducing a new reference gene. The default threshold is 0.15. According to the *V*_*n*_/*V*_*n*__+__1_ ratio, the introduction of a new reference gene can be determined whether to have a significant impact on the standardization factor. If *V*_*n*_/*V*_*n*__+__1_ is less than 0.15, the number of the best reference genes is n; if the ratio is greater than 0.15, the most suitable internal reference number is *n* + 1. In accordance with this principle, our study found that in RT-qPCR experiments with the above mentioned adipose tissue or cells as the experimental object, two reference genes as correction factors are required to correct the experimental data.

In conclusion, the expression stability of 14 endogenous reference genes in adipose tissue and adipocytes of broilers were evaluated. The optimal number of reference genes required for reliable normalization of RT-qPCR data was also determined. Based on our results, the combination of *TBP* and *HMBS* genes is recommended for studies of gene expression in the growth and development of abdominal adipose tissue of broilers, the combination of *PPIA* and *HMBS* genes is effective for studies of gene expression during the proliferation of primary preadipocytes, the *TBP* and *RPL13* are most stable genes for normalization of gene expression during the differentiation of primary preadipocytes, and the *TBP* and *HMBS* are suitable reference genes for the standardization of RT-qPCR data in studies of directly isolated preadipocytes and mature adipocytes. These findings contribute to selection of optimal reference genes required for normalization of RT-qPCR data in the genes function studies in adipose tissue and adipocytes of chickens.

## Data Availability Statement

The original contributions presented in the study are included in the article/Supplementary Material; further inquiries can be directed to the corresponding author/s.

## Ethics Statement

The animal study was reviewed and approved by Institutional Biosafety Committee of Northeast Agricultural University (Harbin, China).

## Author Contributions

WN performed the experiments, analyzed the data, drafted, and edited the manuscript. YW helped design the study, analyzed the data, drafted, and edited the manuscript. PG, XZ, and KZ performed the experiments. HZ and NW participated in designing the study. HL conceived and designed the study, analyzed the data, drafted, and edited the manuscript. All authors read and approved the final manuscript.

## Conflict of Interest

The authors declare that the research was conducted in the absence of any commercial or financial relationships that could be construed as a potential conflict of interest.
