# Supplementary figures and images for: Screening of Reference Genes for RT-qPCR in Chicken Adipose Tissue and Adipocytes
Source: Front Physiol. 2021 May 14;12:676864. doi: 10.3389/fphys.2021.676864 (PMC8160385; doi:10.3389/fphys.2021.676864)

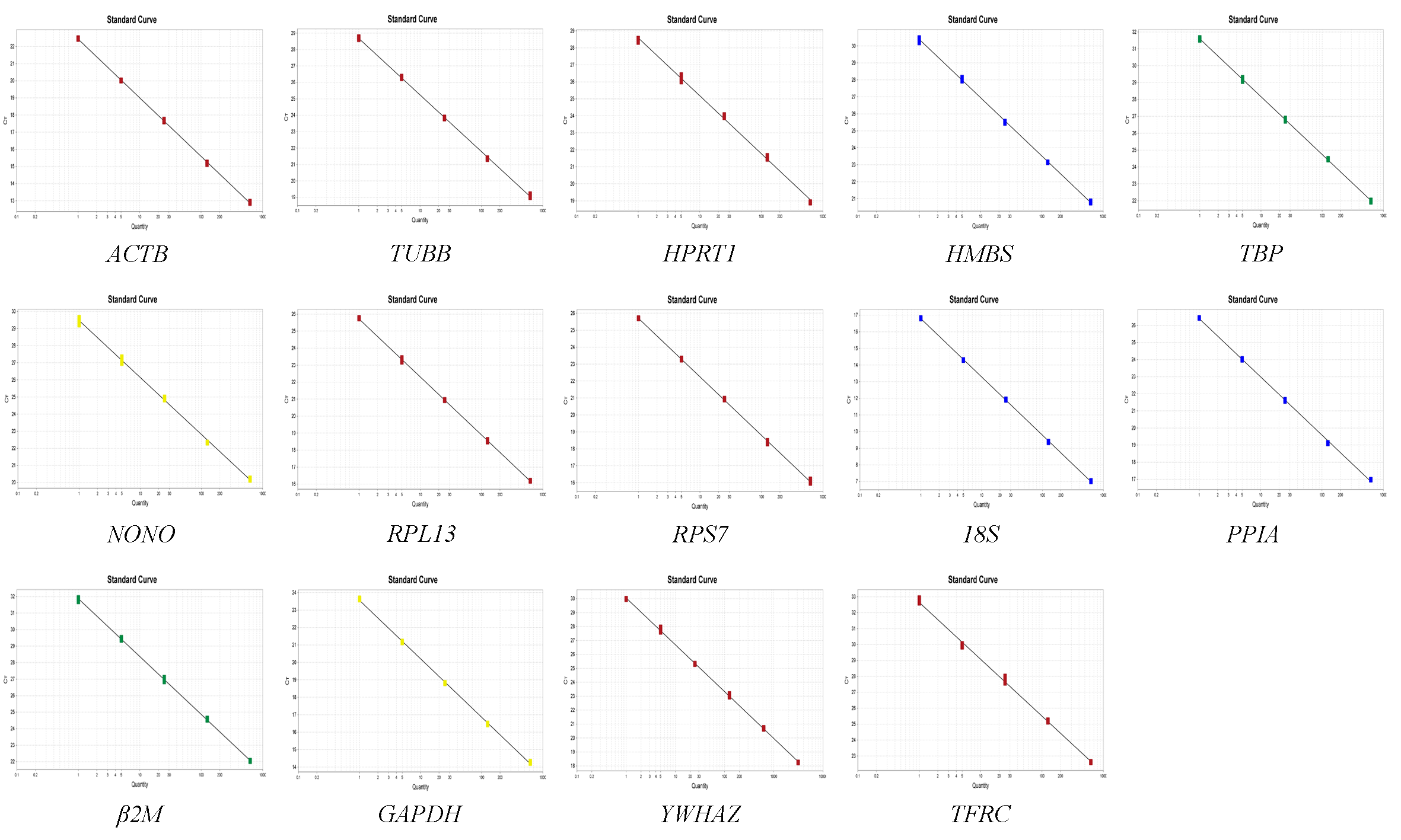

Supplement: Supplementary file 1 [file Image_1.TIF]

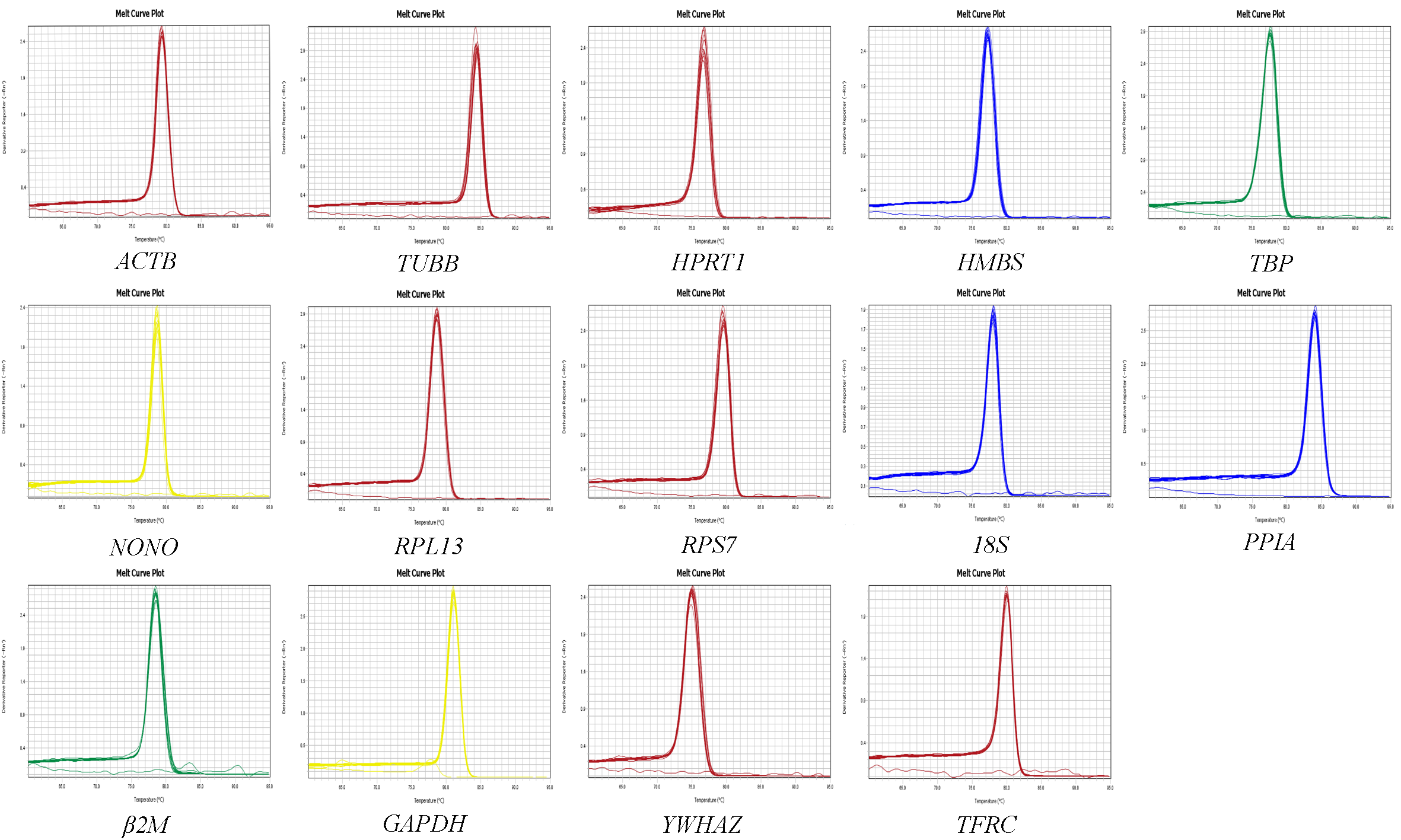

Supplement: Supplementary file 2 [file Image_2.TIF]
