# Supplementary material for: Screening of Reference Genes for RT-qPCR in Chicken Adipose Tissue and Adipocytes
Source: Front Physiol. 2021 May 14;12:676864. doi: 10.3389/fphys.2021.676864 (PMC8160385; doi:10.3389/fphys.2021.676864)
Supplement: Supplementary file 3 [file Table_1.DOCX]

**Supplementary Table 1.** R^2^ and amplification efficiency of the 14 reference genes.

| Gene | R^2^ | Slope | Amplification efficiency（%） | Gene | R^2^ | Slope | Amplification efficiency（%） |
| --- | --- | --- | --- | --- | --- | --- | --- |
| *ACTB* | 1 | -3.421 | 96.025 | *RPS7* | 0.999 | -3.452 | 94.848 |
| *TUBB* | 0.999 | -3.445 | 95.108 | *18S* | 1 | -3.504 | 92.943 |
| *HPRT1* | 0.998 | -3.406 | 96.596 | *PPIA* | 0.999 | -3.408 | 96.518 |
| *HMBS* | 0.999 | -3.435 | 95.492 | *β2M* | 0.999 | -3.501 | 93.048 |
| *TBP* | 1 | -3.425 | 95.887 | *YWHAZ* | 0.999 | -3.36 | 98.419 |
| *NONO* | 0.998 | -3.328 | 99.76 | *GAPDH* | 1 | -3.347 | 98.981 |
| *RPL13* | 1 | -3.41 | 96.44 | *TFRS* | 0.997 | -3.568 | 90.678 |
